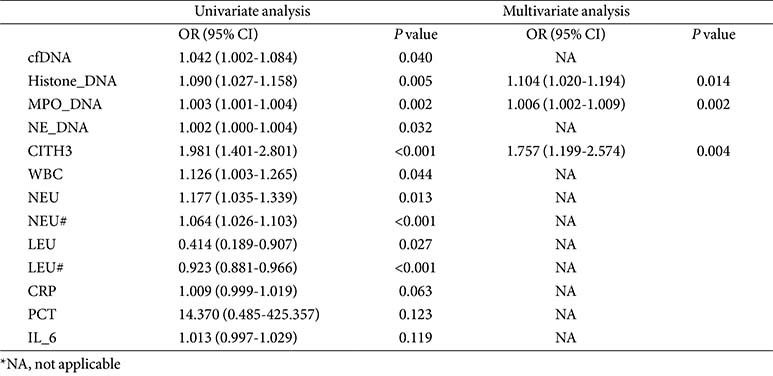# Circulating NET Biomarkers as Predictors of Inflammatory Storm Escalation and Critical Illness in COVID-19

**DOI:** 10.4014/jmb.2509.09004

**Published:** 2025-11-26

**Authors:** Wenjuan Liu, Xin Pan, Ruyue Fan, Ying Yang, Na Sun, Peibin Hou, Zuowang Cheng, Chuanjun Huang, Shuai Liu, Lili Su

**Affiliations:** 1 Department of Pulmonary and Critical Care Medicine, Yantaishan Hospital, Yantai, Shandong, P.R. China; 2 Department of Respiratory and Critical Care Medicine, Shandong Provincial Hospital Affiliated to Shandong First Medical University, Jinan, Shandong, P.R. China; 3 Shandong Center for Disease Control and Prevention, Jinan, Shandong, P.R. China; 4 Department of Clinical Laboratory, Zhangqiu District People’s Hospital Affiliated to Jining Medical University, Jinan, Shandong, P.R. China

**Keywords:** NETs, cfDNA, protein-DNA complexes, CitH3, superior respiratory support, disease severity

## Abstract

COVID-19 demonstrates distinct clinical heterogeneity, ranging from mild symptoms to severe acute respiratory distress syndrome (ARDS). Neutrophil extracellular traps (NETs), which are web-like structures consisting of decondensed DNA adorned with cytotoxic proteins such as myeloperoxidase (MPO) and citrullinated histone H3 (CitH3), play a crucial role in pathogen containment. However, they may also promote immunothrombosis and tissue injury. This research aimed to explore the association between NET formation and the severity of COVID-19. Plasma samples were collected from 99 patients diagnosed between 2022 and 2023. NET remnants were quantified through cell-free DNA (cfDNA), MPO-DNA, neutrophil elastase (NE)-DNA complexes, histone-DNA complexes, and CitH3. The levels of all NET biomarkers were significantly increased in COVID-19 patients and were positively correlated with disease severity. Notably, patients who required mechanical ventilation or high-flow oxygen had significantly higher concentrations of cfDNA, histone-DNA, and CitH3, indicating a strong connection between NETs and respiratory deterioration. Specifically, the combined model incorporating three NETs-related biomarkers demonstrated superior performance in discriminating disease severity, as evidenced by receiver operating characteristic (ROC) analysis. These findings suggest that excessive NET formation contributes to the pathogenesis of COVID-19, potentially via pro-inflammatory and pro-thrombotic pathways. Consequently, the combined model (histone-DNA, MPO-DNA, and CitH3) is identified as a promising biomarker signature for reflecting neutrophil-mediated immunopathology.

## Introduction

Neutrophils, the predominant leukocytes in circulation, serve as initial defenders against microorganism invasion through phagocytosis [1, 2]. This cell, characterized by a brief lifetime and rapid regeneration, contributes to the pulmonary immune response [3]. An essential characteristic of activated neutrophils is their capacity to form neutrophil extracellular traps (NETs), which are web-like structures made of chromatin and comprising the modified histone proteins and granule proteins, including myeloperoxidase (MPO) and neutrophil elastase (NE). The formation of NETs occurs during NETosis, an active cell-death mechanism distinct from apoptosis and necrosis [4].

NETs in pneumonia remain understudied, and they play a double-edged sword role in severe pneumonia. NETs have bactericidal activity, immediately eliminating infections by cytotoxic effects post-capture, or facilitating other neutrophils and phagocytes to phagocytize pathogens, thereby protecting the host. Conversely, NETs may provoke uncontrolled amplification of inflammatory cascades, resulting in lung tissue injury, and immunothrombosis. Current evidence suggests their dual functionality in both host defense and disease development. However, the precise molecular mechanisms by which NETs influence the pathophysiology of severe pneumonia and their temporal functions in the progression of the disease remain inadequately elucidated.

Until recently, neutrophils and NETosis were not regarded as significant contributors to respiratory viral infections [5]. Growing evidences demonstrates that neutrophils may have both beneficial and detrimental impacts during viral infections. Take influenza virus infection as an example. Elevated circulating levels of NETs are correlated with poor prognosis after influenza A infection, whereas increased NETs levels in bronchoalveolar lavage fluid correspond with lung disease [6]. Our previous studies highlighted neutrophils activation and NETs formation as the most indicative characteristics of severe influenza [7]. Several studies indicated that neutrophil depletion exacerbates the severity of these diseases in mice infected with H3N2 influenza [8, 9]. Other studies have shown that limiting neutrophil influx after influenza A virus infection reduces the severity of pulmonary damage [10, 11]. Arginine-rich histones can inhibit the uptake and replication of influenza virus by directly interacting with virus particles, thereby safeguarding the organism [6]. These two apparently contradictory statements suggest that the function of neutrophils and their exosomal production in respiratory viral infections remains unclear. Whether these specific viruses are hijacking neutrophils for their own benefit or temporal variations in the antiviral response needs to be elucidated.

Millions of patients worldwide were affected by the coronavirus disease in 2019 (COVID-19). Major progress has been accomplished regarding the characterization of neutrophils in patients with COVID-19. COVID-19 parallels influenza, presenting a clinical range from mild upper respiratory symptoms to acute respiratory distress syndrome necessitating ventilatory support [12]. The excessive recruitment and activation of neutrophils, together with NETosis, are risk factors for acute lung injury (ALI) or acute respiratory distress syndrome (ARDS) caused by SARS-CoV-2 infection [13-15]. Emerging research indicates that NETs may serve as both a predictive biomarker for clinical stratification and a promising therapeutic target, possibly enabling novel immunomodulatory interventions for this life-threatening condition [16, 17]. A judicious equilibrium of NETs formation may emerge as a potential avenue for the treatment of severe COVID-19 in the future. The severity of illness is intimately linked to the selection of timely and suitable respiratory support, which is crucial for the early identification of critically ill patients and the reduction of mortality via appropriate intervention. Nevertheless, research on NETs and the selection of respiratory support strategies after the assessment of illness severity is still lacking.

This research aims to determine various plasma NETs indicators in hospitalized patients and determine their association with disease severity and the highest required respiratory support strategy, and their interaction with inflammatory markers. These data provide insights for research and early intervention or treatment of pulmonary infectious diseases.

## Materials and Methods

### Study Population and Sample Collection

Between December 2022 and July 2023, a total of 99 adult COVID-19 patients and 38 healthy controls were recruited from the Department of Pulmonary and Critical Care Medicine at Shandong Provincial Hospital in Jinan, China. The reverse real-time polymerase chain reaction (RT-PCR) examination of throat samples confirmed the presence of SARS-CoV-2 infection in all patients. Patients were diagnosed with pneumonia through chest computed tomography (CT). In accordance with the 10th Diagnosis and Treatment Plan for novel coronavirus Infection released by the National Health Commission of China, moderate pneumonia was characterized by high fever or respiratory tract symptoms (cough, shortness of breath, etc), accompanied by imaging findings indicative of pneumonia. The defining features of severe pneumonia included dyspnea, tachypnea (respiratory rate ≥ 30/min), hypoxemia (SpO_2_ ≤ 93% or PaO_2_/FiO_2_ ratio ≤ 300), and/or extensive lung infiltrates (> 50%) appearing within 24–48 h; alternatively, it manifested as respiratory failure, septic shock, and/or multiple organ dysfunction syndrome (MODS)/failure. The inclusion criteria required that all enrolled patients show no evidence of bacterial or fungal infections, as well as no history of chronic inflammatory diseases. Based on the diagnostic criteria described above and previously reported [18], 57 patients were diagnosed with severe disease, whereas 42 patients were classified with moderate illness. Fatal outcomes were defined as death from any cause within 60 days of COVID-19 hospitalization. A control group included 38 healthy individuals without signs of viral infection. All samples were collected within 24 h of patient admission, processed within 2 h, centrifuged at 1,500 rpm for 10 min at 20°C, and stored as plasma at -80°C until thawed for testing.

### Chest CT Protocols and Image Analysis

All images were acquired using a multi-detector HiSpeed Dual CT scanner, covering the region from the upper thoracic inlet to the inferior costophrenic angle. Reconstructed at 0.6-mm slice thickness with identical increments, the chest CT images were independently reviewed by three experienced radiologists (5+ years); discrepancies were resolved through consensus discussion. The area of interest was manually delineated by marking the lesion’s most confined area (*i.e.*, the area of highest intensity) on CT images. The predominant chest CT findings in COVID-19 patients included ground-glass opacity (GGO), consolidation, reticulation (with/without septal thickening), linear bands, bronchial wall thickening, nodules, bronchiectasis, and interlobar pleural traction [19, 20].

### Measurement of Cell-Free Deoxyribonucleic Acid (cfDNA)

The cfDNA levels in the plasma were quantified using the Quant-iT PicoGreen double-stranded DNA (dsDNA) assay in accordance with the manufacturer’s instructions (Thermo Fisher Scientific, USA). After loading standards/plasma (50 μl) into black 96-well plates, 50 μl Quant-iT PicoGreen working solution was added to each well. The plates were incubated at 20°C for 5 min in light-protected conditions, then fluorescence intensity (emission: 485 nm) was quantified on a Varioskan Flash microplate reader (Thermo Fisher Scientific). Serial dilutions created a 5-point standard curve ranging from 1 ng/ml to 1 μg/ml, which was used to determine sample DNA concentrations.

### The Quantification of Histone-DNA and Myeloperoxidase (MPO)-DNA Complexes

Flat-bottom 96-well plates were coated with anti-histone antibody (Roche Cell Death ELISA^PLUS^ Kit, SE) and anti-MPO antibody (Abcam, UK) at 1:500 dilution. Plasma samples (20 μl) were mixed with 80 μl incubation buffer containing 5% HRP-conjugated anti-DNA antibody (Roche Cell Death Detection ELISA Kit, SE) and incubated (2 h, 20°C). After adding 3-ethylbenzothiazoline-6-sulphonic acid substrate, absorbance was measured at 405 nm. To minimize inter-assay variability, sample optical density (OD) values were normalized to a reference plasma OD (OD index = sample OD/reference OD).

### Measuring Neutrophil Elastase (NE)-DNA and Citrullinated Histone H3 (CitH3) Concentrations

The concentrations of NE-DNA (mlbio) and CitH3 (J&l Biological) in the plasma samples were measured according to the manufacturer’s instructions. To quantify NE-DNA complexes, plates were coated with an anti-NE antibody. After blocking, the plates were incubated with diluted plasma samples. The bound DNA was detected using a peroxidase-conjugated anti-DNA antibody, followed by the addition of TMB substrate for color development; the absorbance was then measured at 450 nm. For CitH3, a sandwich ELISA was conducted: plates pre-coated with an anti-CitH3 antibody were sequentially incubated with plasma samples, a biotinylated detection antibody, streptavidin-HRP, and TMB substrate. All the samples were diluted 2 times prior to detection. The protein levels were quantified against standard curves.

### Independent Prognostic Analysis

Univariate and multivariate Cox regression analyses were performed to evaluate the independent prognostic capability of the signature. Receiver operating characteristic (ROC) curves were generated with the R packages (‘pROC’), and the corresponding area under the curve (AUC) values were computed.

### Statistical Analysis

Statistical analyses used GraphPad Prism 5.0, applying Student’s *t*-test, Mann-Whitney U test, chi-square test, or Fisher's exac*t* test as appropriate. *P < 0.05, **P < 0.01 and ***P < 0.001 represent significant differences.

## Results

### Demographics and Clinical Characteristics of Patients with COVID-19

**Fig. 1:**
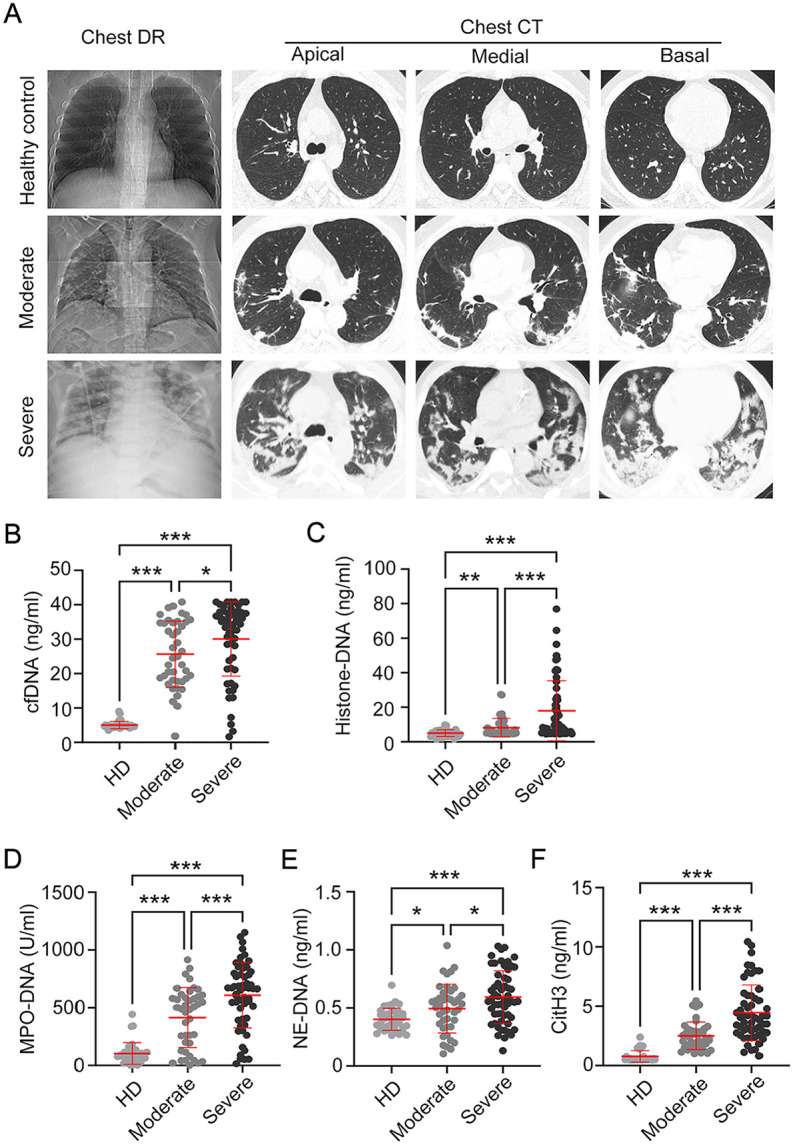
Increased plasma levels of neutrophil extracellular traps in patients with COVID-19 were associated with disease severity. (**A**) Chest computed tomography (CT) scan features of COVID-19. Chest CT from moderate patients showed bilateral scattered ground glass opacities. And the severe patients showed confluent and predominantly patchy ground glass opacities with pronounced peripheral distribution, and partial consolidation. (**B-E**) Plasma levels of (**B**) cell-free deoxyribonucleic acid (cfDNA), (**C**) histone-DNA, (**D**) myeloperoxidase (MPO)-DNA, (**E**) neutrophil elastase (NE)-DNA, and (**F**) citrullinated histone H3 (CitH3) in the patients with COVID-19 and healthy donors (HD). Data are displayed as the mean ± SD. Statistical significance was determined by an unpaired *t* test. *P < 0.05, **P < 0.01, ***P < 0.001.

A total of 137 participants were included in this study, comprising 99 COVID-19 patients and 38 healthy individuals. All COVID-19 patients enrolled had at least one comorbidity, with the most prevalent disease following pneumonia being hypertension. Typical chest CT imaging was shown in Fig. 1A. Imaging studies conducted at the time of admission can assist in risk stratification of patients with COVID-19. Bilateral lungs showed multiple linear band-shaped ground-glass opacities in moderate patients on chest CT. Conversely, severe patients exhibited bilateral multifocal consolidations with characteristic peripheral predominance on chest CT. And the lesions were predominantly localized across the whole lung area, commonly referred to as the “white lung” sign. The total leukocyte counts, neutrophil counts, and percentage of neutrophils were markedly elevated in severe patients compared to moderate patients, whereas lymphocyte counts and the proportion of lymphocytes were diminished. Both serum albumin and PaO_2_/FiO_2_ ratios were significantly decreased in severe patients compared to moderate groups. Hence the highest-level respiratory support strategy was implemented and systematically summarized. Among all patients, 92.93% (92 of 99) required at least one type of supplemental oxygen support. Among severe cases, 50.88% (29/57) required advanced respiratory support, such as high-flow nasal cannula oxygen therapy (HFNC) or mechanical ventilation support, with 14.04% in-hospital mortality observed. The clinical characteristics of all participants are summarized in Table 1.

### Elevated Plasma NETs Correlate with Increased COVID-19 Severity

To investigate and distinguish the functions of NETs following infection, plasma was extracted from COVID-19 patients and healthy donors to analyze various markers of the formation process of NETs (also referred to NETosis). Soluble NETs remnants can exist in the form of cfDNA. Compared with healthy donors, COVID-19 patients exhibited elevated plasma cfDNA concentrations, which were highest in the severe group (Fig. 1B). And all specific for NETs remnants, histone-DNA, MPO-DNA, and NE-DNA complexes (Fig. 1C-1E) displayed similar profiles in COVID-19 patients and healthy controls. CitH3 is a marker of NETosis, and the severely infected patients had higher levels of CitH3 in the plasma than moderate patients and healthy controls (Fig. 1F). Together, these data demonstrate that plasma NET remnants levels correlate with COVID-19 severity, suggesting their pathogenic role in disease progression.

### Elevated NETs Levels Correlated with Superior Respiratory Support in COVID-19

**Fig. 2:**
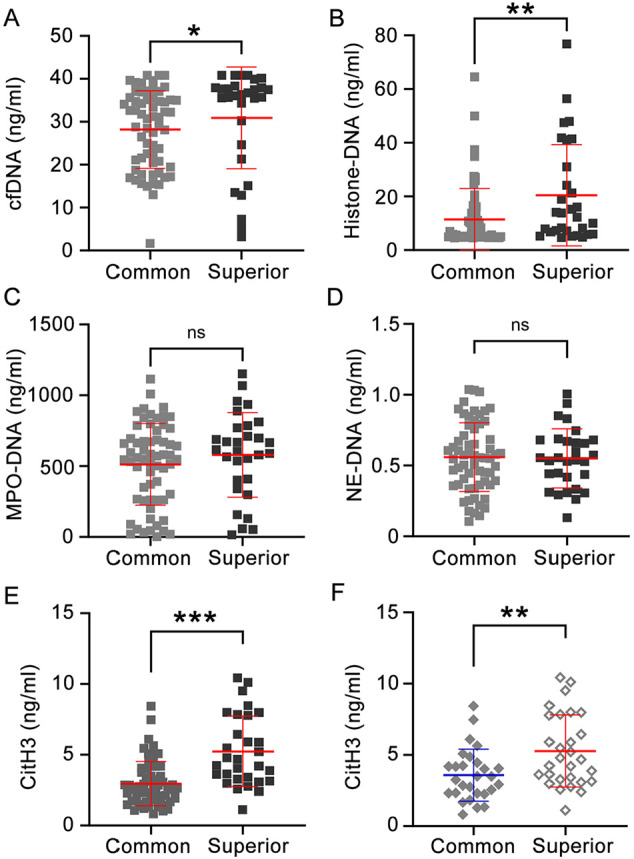
Elevated neutrophil extracellular traps levels were correlated with superior respiratory support in COVID-19. Plasma samples were grouped by specific respiratory support strategies (Common, oxygen provided via nasal cannula or face mask; Superior, high-flow nasal cannula oxygen therapy or mechanical ventilation) and analyzed for (**A**) cfDNA, (**B**) histone-DNA, (**C**) MPO-DNA, (**D**) NE-DNA, and (**E**) CitH3. Groups were compared by Mann-Whitney *U* test; *P < 0.05, **P < 0.01, and ***P < 0.001; *ns*, not significant. Square symbols represent individual plasma samples from patients. Gray: Common; Black: Superior. (**F**) Plasma levels of CitH3 in severe patients with different respiratory support strategies. Statistical significance was determined by an unpaired *t* test. **P < 0.01. Diamond symbols represent individual plasma samples from patients. Solid: Common; Hollow: Superior.

The disease severity can be reflected by the type of respiratory support required; therefore, we next assessed the clinical status corresponding to each accessible plasma sample. We compared samples from individuals necessitating advanced respiratory support (*e.g.*, HFNC or mechanical ventilation, *n* = 30 samples) with samples from patients receiving less intensive support (oxygen provided via nasal cannula or face mask, *n* = 62 samples). As compared with patients who received conventional oxygen therapy, patients requiring mechanical ventilation or HFNC had significantly higher levels of cfDNA (Fig. 2A), histone-DNA (Fig. 2B) and CitH3 (Fig. 2C), but not MPO-DNA (Fig. 2D) and NE-DNA (Fig. 2E). Elevated CitH3 levels were specifically observed in severely ill COVID-19 patients requiring escalated respiratory support (Fig. 2F). Collectively, these data indicate higher levels of circulating NETs were associated with more severe oxygenation deterioration.

### Circulating NETs Exhibited Significant Positive Correlations with PCT

**Fig. 3:**
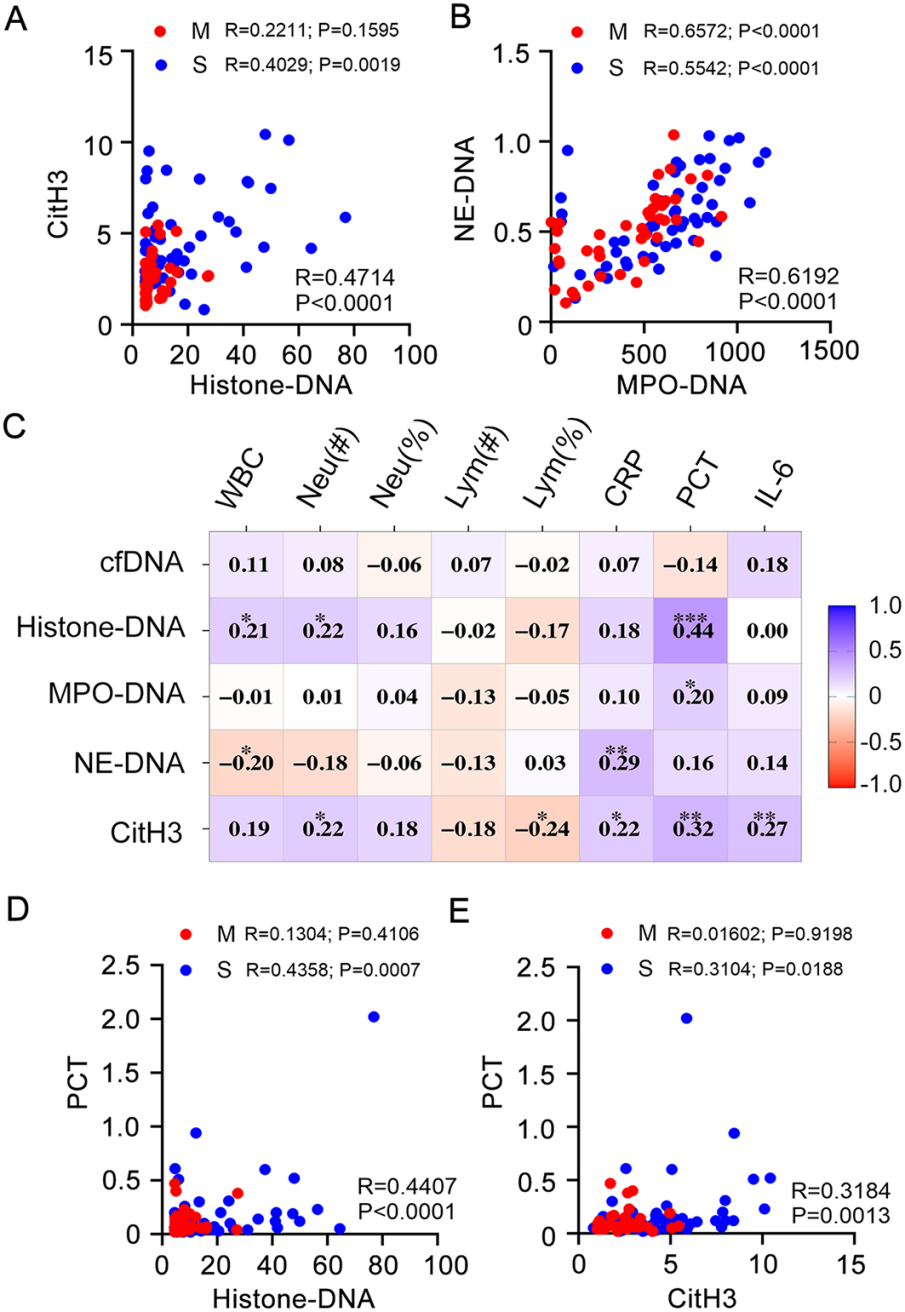
Association between neutrophil extracellular traps and clinical parameters in patients with COVID-19. (**A-B**) Correlation analysis and scatter plot of the neutrophil extracellular traps remnants in the plasma of patients with COVID-19. (**C**) Correlation-based heat-map demonstrating the association between the measured neutrophil extracellular traps remnants and clinical parameters in patients with COVID-19. Blue represents positive correlation, and red represents negative correlation. (**D-E**) Correlation analysis between procalcitonin expression levels and key neutrophil extracellular traps remnants (**D**) histone-DNA and (**E**) CitH3. A Pearson correlation test was used for the association, *P < 0.05, **P < 0.01, ***P < 0.001.

Numerous laboratory markers, including neutrophils, lymphocytes, CRP, procalcitonin (PCT), and IL‐6, have been reported to be related to the morbidity and mortality of COVID-19. Thus, the association between circulating NETs and laboratory parameters was further assessed. All available samples (*n* = 99) underwent subsequent correlation analyses. Significant correlations linked histone-DNA to CitH3 (r = 0.4714, *p* < 0.0001), and similarly connected MPO-DNA with NE-DNA (r = 0.6192, *p* < 0.0001) (Fig. 3A-3B). Pearson correlation analyses were conducted between NETs-related biomarker measurements and inflammatory indicators, as shown in Fig. 3C. The results were also listed in Tables S1 and S2. Additionally, scatter plots highlighted results where correlation coefficients exceeded |0.3| and were statistically significant (*p* < 0.05). Results showed that histone-DNA and CitH3 exhibited significant positive correlation with PCT, respectively (Fig. 3D and 3E). Of note, although integrated data from all influenza patients suggested significant correlations, no such correlation was found in moderate patients between CitH3 and histone-DNA, PCT and histone-DNA, or PCT and CitH3 (Fig. 3A, 3D, and 3E). In summary, these data indicate a potential correlation between plasma NET levels and dysfunctional inflammatory response.

### Circulating NETs as Putative Biomarkers for Predicting Disease Severity

**Fig. 4:**
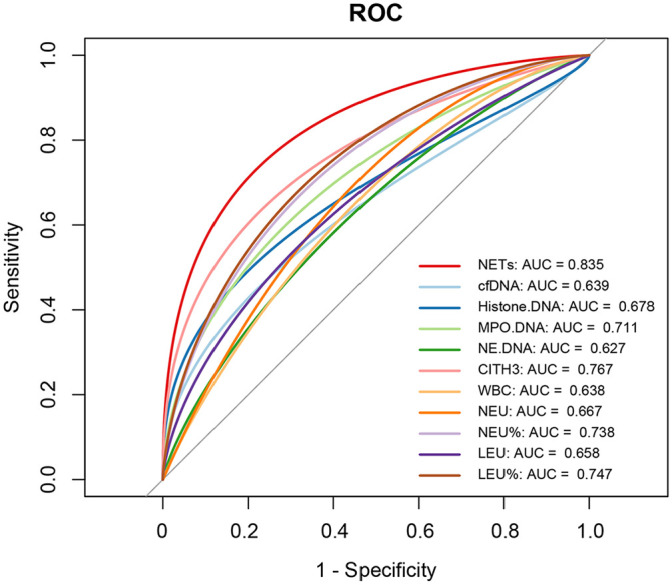
Receiver-operator characteristic (ROC) curves of neutrophil extracellular traps remnants and main clinical parameters for predicting disease severity. NETs represent the diagnostic efficacy of the combination of three significant indicators in multivariable logistic regression.

Given the central role of neutrophils and NETs in COVID-19 pathogenesis, we posit that quantification of their associated biomarkers could reflect clinical disease progression. The clinical parameters and NETs-related indicators for all population groups are presented in Table S3. Univariate and multivariate logistic regression analyses identified histone-DNA, MPO-DNA, and CitH3 as significant predictors among various NETs-related biomarkers and clinical parameters. Histone-DNA (Univariate OR: 1.090, 95% CI: 1.027~1.158, *P* = 0.005; Multivariate OR: 1.104, 95% CI: 1.020~1.194, *P* = 0.014), MPO-DNA (Univariate OR: 1.003, 95% CI: 1.001~1.004, *P* = 0.002; Multivariate OR: 1.006, 95% CI: 1.002~1.009, *P* = 0.002), and CitH3 (Univariate OR: 1.981, 95% CI: 1.401~2.801, *P* < 0.001; Multivariate OR: 1.757, 95% CI: 1.199~2.574, *P* = 0.004) all demonstrated consistent significance in both models (Table 2). ROC curves were used to evaluate the predictive value of NETs-related biomarkers and clinical parameters. The results indicated an AUC value of 0.767 for CitH3 in predicting severity, exceeding the AUC values of all clinical parameters for this prediction. Furthermore, the combined model incorporating three NETs-related biomarkers achieved an AUC of 0.835, significantly outperforming models based on other indicators, confirming its superior ability to distinguish disease severity (Fig. 4). Collectively, these data suggest circulating NETs demonstrate potential for predicting outcomes and implicate inflammatory imbalance in poor COVID-19 prognosis.

## Discussion

This research focused on understanding how NETs impact disease progression and clinical outcomes in individuals admitted to the hospital due to COVID-19. Our study revealed a new observation: patients in need of advanced respiratory assistance exhibited markedly increased plasma concentrations of NETs. In addition, our results supported prior evidence suggesting that elevated NET concentrations in the bloodstream are closely linked to disease severity and inflammatory response, which suggested that early and timely monitoring of NETs is beneficial for evaluating the extent of inflammatory response and the status of immune function in these patients. Finally, we have demonstrated that on the day of patient admission, the plasma levels of CitH3 and MPO-DNA have a relatively significant advantage in predicting the disease severity. These results may offer new perspectives for optimizing the clinical application of antibiotics and immunomodulators, ultimately contributing to better patient outcomes.

Earlier investigations have shown that fragments derived from neutrophil immune responses-such as extracellular nucleic acids and chromatin-bound particles tend to accumulate in individuals affected by COVID-19 [21, 22]. Building on these observations, our study not only verified this increase but also uncovered a link between the presence of these immune byproducts and the clinical decision to implement advanced respiratory support. The results suggested that increased levels of circulating NETs were correlated with local pulmonary dysfunction. This finding may be attributed to several reasons. One notable explanation is that a considerable proportion of critically ill patients succumbed to ARDS [23-25]. Acute inflammation, both local and systemic,is a key feature of ARDS and plays a critical role in damaging both the lung epithelium and endothelium. During this process, neutrophils move from the pulmonary circulation into the airway spaces, where they can release various harmful substances [26]. In our cohort, 14.04% (8/57) of patients diagnosed with ARDS succumbed within a 60-day period. This indicates that ARDS played a more pivotal role in fatal outcomes among critically ill individuals than broader respiratory insufficiency. Accordingly, measurements of extracellular DNA in the bloodstream serve as more accurate biomarkers for COVID-19-related ARDS. Secondly, an elevation in plasma NETs levels may result from the hyperactivation of circulating neutrophils and/or the compromise of the alveolar-capillary barrier. In clinical practice, infection with SARS-CoV-2 often presents alongside vascular disorders in the lungs, such as the development of embolic events [27, 28]. Vascular injury and thrombosis are significant mechanisms of air-blood barrier disruption induced by COVID-19. The extent of clot-related complications, including microvascular thrombosis and deep venous clot formation, has shown a strong association with the concentration of specific indicators linked to NETs [29]. In ARDS, the formation of NETs may disrupt the equilibrium between inflammatory responses and coagulation processes [30]. Long-term exposure to this elevated inflammatory equilibrium leads to pathological repair, characterized by persistent damage and repeated repair of normal tissues, which eventually leads to tissue fibrosis, thickening of the air-blood barrier, and obstruction of gas exchange between the alveoli and the microcirculation. These two scenarios may indicate the progression of multi-organ damage driven by widespread inflammatory activity. In conclusion, elevated NET remnant levels in patients with progressive pulmonary function decline further substantiate their role as indicators of severe lung involvement.

Notably, in our analysis of various biomarkers linked to NETs, only cfDNA, histone-DNA and CitH3 showed significant increases in patients requiring an advanced respiratory support strategy, while more targeted indicators like MPO-DNA and NE-DNA remained comparatively unchanged. Cell-free DNA and nucleosome-associated material are not exclusively derived from neutrophils; they may also be released by other immune cells like eosinophils and mastocytes, as well as by structural tissue cells during programmed cell death or unregulated cellular breakdown [31]. Therefore, DNA fragments found outside cells in the blood of COVID-19 patients could stem from multiple cellular sources, including vascular lining cells, airway epithelium, and immune cells beyond neutrophils. These findings align with the dominant perspective that respiratory impairment in COVID-19 is driven by a combination of contributing factors [32]. Despite the uncertainty regarding the cellular origin, the plasma levels of cfDNA and histone-DNA are more likely indicative of pulmonary inflammation and tissue damage.

Histone H3 can undergo chemical alteration after protein synthesis, resulting in a modified form known as citrullinated H3 (CitH3). During NETs formation, histones are expelled from the nuclei of neutrophils and subsequently citrullinated through the enzymatic actions of protein arginine deiminase 4 (PAD4) and the calcium-dependent enzyme peptidyl arginine deiminase 2 (PAD2) [33]. This modification leads to the extrusion of CitH3 into the extracellular environment. Recent studies have linked CitH3 levels to the severity of sepsis[34] and suggested its potential as a therapeutic target for endotoxic shock [35]. Histone H3 is recognized for inducing thrombocytopenia and thromboembolic events by activating and aggregating platelets [36-38], and it exerts potent cytotoxic effects via the activation of apoptosis. Nevertheless, the exact function of CitH3 in ARDS caused by COVID-19 has yet to be fully elucidated. This study demonstrates that CitH3 adversely impacts disease progression and serves as a detrimental prognostic indicator.

A broad range of clinical biomarkers, such as total leukocyte levels, lymphocyte and neutrophil measurements, along with inflammatory markers like CRP, PCT, and IL-6, have been thoroughly explored in prior research and linked to both the progression and fatal outcomes of COVID-19 [39]. This research focused on plasma levels of NETs and observed that the plasma levels of CitH3 and MPO-DNA could serve as a more reliable indicators for recognizing individuals at risk of severe progression than traditional inflammatory markers such as white cell counts, IL-6, CRP, and PCT. And histone-DNA and CitH3 exhibited significant positive correlation with PCT, respectively. Because PCT elevation is typically a result of concurrent bacterial involvement, its usefulness in evaluating the severity of illness shortly after initial SARS-CoV-2 exposure may be significantly reduced [40]. Therefore, circulating CitH3 and histone-DNA could act as standalone biomarkers for predicting the likelihood of critical disease progression in individuals infected with COVID-19.

There are several constraints to our research. Firstly, it is still unclear whether the detected NET remnants play a direct role in worsening the disease or are merely secondary outcomes of the intense inflammatory response observed in patients. Secondly, whether changes in NETs are specific to SARS-CoV-2 infection remains unknown. Similar to several investigations, this research compared healthy or non-severe individuals to severe COVID-19 patients. Although this does not invalidate findings in SARS-CoV-2 infection, their conclusions may overstate a specific cell or pathway’s role as unique to COVID-19 pathogenesis when it mirrors responses seen in conditions like sepsis from other causes. Thirdly, it is important to note that the association between elevated NETs and poor oxygenation may be confounded by overall disease severity. Future studies incorporating detailed and serial assessments of disease severity are needed to confirm that NETs contribute to lung injury independently of the overall clinical state. Finally, the majority of patients enrolled in this study received combination therapies, which included corticosteroids among other medications, which could affect neutrophil number and the generation of NETs. Future research needs to clearly demonstrate that, after eliminating confounding factors, using plasma levels of NETs to assist in predicting and evaluating the disease severity of COVID-19 may have a positive effect.

## Conclusion

Our research demonstrated a significant correlation between the presence of circulating NETs fragments and the severity of illness, as well as systemic inflammatory activity. Notably, patients who needed escalated forms of respiratory assistance showed markedly higher levels of NETs in their blood. Moreover, the combined model incorporating three NETs-related biomarkers demonstrated superior performance in discriminating disease severity. These outcomes may offer valuable directions for optimizing clinical decisions, particularly in the use of antimicrobial agents and immune-modulating therapies, ultimately enhancing patient recovery and outcomes.

## Supplemental Materials

Supplementary data for this paper are available on-line only at http://jmb.or.kr.



## Figures and Tables

**Table 1:** Patient characteristics by disease severity.

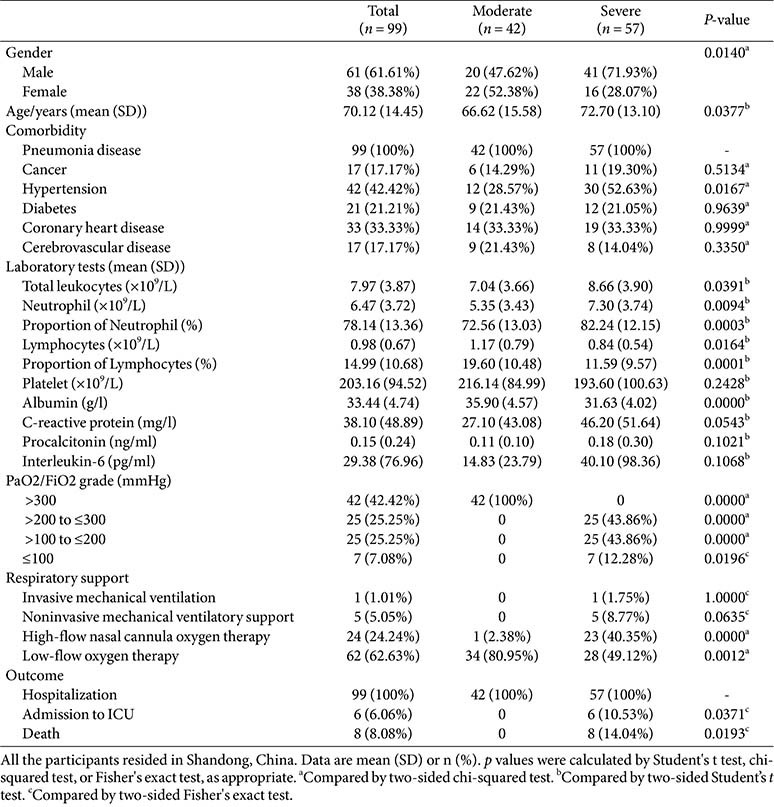

**Table 2:** Univariate and multivariate logistic regression.