# Supplementary material for: Circulating NET Biomarkers as Predictors of Inflammatory Storm Escalation and Critical Illness in COVID-19
Source: J Microbiol Biotechnol. 2025 Nov 26;35:e2509004. doi: 10.4014/jmb.2509.09004 (PMC12685590; doi:10.4014/jmb.2509.09004)
Supplement: Supplementary file 1 [file jmb-35-e2509004-supple.pdf]

J. Microbiol. Biotechnol. 2025. 35: e2509004
https://doi.org/10.4014/jmb.2509.09004

Supplementary Tables

Circulating NET biomarkers as predictors of inflammatory storm escalation and critical illness in COVID-19

Table S1. Relationship between biomarkers of NETs and clinical clinical parameter during acute COVID-19

r	cfDNA	Histone-DNA	MPO-DNA	NE-DNA	CitH3
WBC	0.110444094	0.211612428	-0.014904316	-0.204971728	0.187212421
Neu(#)	0.083048865	0.217861469	0.005498048	-0.184707674	0.218052136
Neu(%)	-0.063673842	0.162564036	0.04155376	-0.056750402	0.17985361
Lym(#)	0.073599417	-0.018165783	-0.133231692	-0.133046283	-0.183412424
Lym(%)	-0.022084342	-0.173513645	-0.047881764	0.030153184	-0.236451621
CRP	0.071204511	0.179479953	0.103766684	0.287015759	0.22100373
PCT	-0.144213481	0.440726062	0.204077778	0.155308751	0.318425326
IL-6	0.176695726	-0.001740591	0.091864077	0.143014032	0.270458263

Table S2. Relationship between biomarkers of NETs and clinical clinical parameter during acute COVID-19

p	cfDNA	Histone-DNA	MPO-DNA	NE-DNA	CitH3
WBC	0.276470717	0.035496296	0.883589213	0.041830311	0.063521368
Neu(#)	0.413788802	0.030291633	0.956926782	0.06721111	0.030143566
Neu(%)	0.531225482	0.107909067	0.682994571	0.576886704	0.07485536
Lym(#)	0.469073566	0.858356676	0.188620116	0.189241541	0.069186185
Lym(%)	0.828230031	0.085875646	0.637899963	0.76701821	0.018457921
CRP	0.483697614	0.075471655	0.306726349	0.003972147	0.027928409
PCT	0.154406334	4.98459E-06	0.042750841	0.124782092	0.001317702
IL-6	0.080193216	0.986357856	0.365814222	0.157899916	0.006779125

Table S3. Test results for enrolled subjects

No	Severity	cfDNA	Histone-DNA	MPO-DNA	NE-DNA	CITH3	WBC	NEU	NEU%	LEU	LEU%	CRP	PCT	IL-6
1	S	40.829	7.9125	697.5388	527.7735	3.644422	5.31	4.33	81.6	0.66	12.4	11.65	0.03	1.5
2	S	37.648504	5.2539	408.7976	311.2605	2.411638	4.79	3.28	68.4	1.19	24.9	1.5	0.03	6.82
3	S	13.023046	5.1906	787.1118	549.6435	4.01866	5.93	4.43	74.7	1.16	19.6	10.23	0.02	50.64
4	M	40.829	5.2539	459.38	219.4065	2.070421	5.82	3.99	68.5	1.22	21	5.85	0.05	8.17
5	S	12.88273	21.3321	672.2476	437.013	2.774869	13.76	12.99	94.4	0.41	3	73.19	0.2	2.5
6	S	14.964084	4.8108	301.31	242.37	1.674169	19.9	18.25	91.7	1.03	5.2	21.29	0.11	1.5
7	S	34.28092	18.6102	545.7916	357.1875	3.490324	7.8	6.72	86.1	0.51	6.5	9.77	0.04	4.65
8	S	39.940332	5.4438	565.8138	446.8545	3.083065	13.01	11.15	85.7	1.29	9.9	61.61	0.14	55.18
9	S	35.555457	18.99	786.058	680.8635	1.123819	6.43	4.49	69.8	1.3	20.2	10.19	0.07	1.5
10	S	37.706969	6.7731	1151.7266	937.836	2.984002	8.68	7.21	83.1	0.93	10.7	19.38	0.1	27.75
11	S	37.496495	10.0647	604.8044	656.8065	4.67908	8.61	7.15	83.1	0.87	10.1	0.45	0.05	3.8
12	M	24.505572	5.2539	750.2288	793.494	1.92733	5.74	3.88	67.6	1.34	23.3	11.85	0.17	19.7
13	S	38.116224	4.6842	853.5012	906.1245	4.051681	6.53	5.07	77.6	0.97	14.9	13.1	0.04	11.77
14	M	18.507063	4.6209	488.8864	517.932	1.277917	6.76	5.44	80.4	1.22	80.4	0.31	0.05	1.5
15	S	1.645757	5.6337	1009.4636	1020.942	6.087976	2.31	2.1	90.9	0.16	6.8	4.85	0.09	3.35
16	M	15.385032	4.8108	641.6874	848.169	5.075332	8.39	7.01	83.6	0.71	8.5	11.83	0.05	3.2
17	S	40.829	5.9502	935.6976	851.4495	9.52216	9.97	9.18	92.1	0.25	2.5	165.4	0.51	658
18	S	23.768913	5.1273	652.2254	508.0905	1.288924	4.78	3.56	74.5	0.8	16.7	16.93	0.04	2.54
19	S	39.039971	10.2546	684.8932	711.4815	3.501331	7.07	5.78	81.8	0.89	12.6	35.95	0.02	2.09
20	S	40.829	11.0775	849.286	1031.877	2.554729	7.19	6.49	90.3	0.38	5.3	28.85	0.09	9.54
21	M	20.798891	5.1906	614.2886	672.1155	1.200868	4.06	3.37	83	0.21	11.3	1.3	0.04	1.5
22	S	40.139113	8.229	554.222	533.241	4.78915	4.72	4.19	88.7	0.42	8.8	28.76	0.26	354
23	M	39.145208	5.0007	501.532	335.3175	3.072058	4.55	3.53	77.5	0.72	15.9	56.16	0.08	1.5
24	M	18.717537	4.6209	840.8556	813.177	2.136463	5.36	4.43	82.6	0.79	14.7	4.85	0.02	1.5
25	S	16.414016	4.6209	885.1152	365.9355	2.334589	7.93	6.54	82.5	0.94	11.9	60.77	0.03	7.07
26	S	35.473606	13.8627	537.3612	529.9605	5.482591	12.95	10.76	83.1	0.97	7.5	55.8	0.06	11.4

27	S	28.773517	8.4189	513.1238	494.9685	2.301568	7.2	6.59	91.5	0.52	7.2	55.27	0.12	115.6
28	M	15.57212	10.6344	595.3202	645.8715	1.465036	6.46	5.29	81.9	0.89	13.7	2.38	0.06	2.79
29	S	24.657581	15.2553	401.421	333.1305	3.424282	14.42	13.64	94.6	0.27	1.9	21.98	0.1	61.61
30	M	33.731349	8.0391	673.3014	567.1395	2.752855	8.59	6.53	76	1.49	17.3	19.23	0.23	102.6
31	S	32.6439	4.7475	669.0862	617.4405	1.343959	6.76	3.79	56.1	2.22	32.8	1.94	0.16	69.01
32	M	19.454196	10.8243	584.7822	571.5135	1.850281	10.77	8.08	75	2.21	20.5	2.9	0.07	1.5
33	M	20.459794	4.7475	609.0196	562.7655	1.04677	4.74	2.77	58.5	1.36	28.7	0.4	0.09	3.24
34	S	21.313383	4.6842	156.9394	262.053	2.576743	6.98	6.51	93.3	0.27	3.9	42.23	0.61	1.5
35	S	37.08724	7.6593	672.2476	861.291	2.246533	5.34	4.15	77.7	0.76	14.2	1.08	0.02	1.87
36	S	21.114602	4.5576	350.8386	386.712	2.312575	4.48	3.58	79.8	0.65	14.5	48.9	0.06	39.88
37	M	17.747018	4.6209	659.602	1037.3445	1.729204	11.31	8.6	76	1.32	11.7	177.02	0.47	85.65
38	S	31.053652	4.5576	694.3774	865.665	2.928967	10.07	8.79	87.3	0.79	7.8	125.36	0.2	57.13
39	M	35.485299	5.1273	78.9582	105.6825	1.145833	13.06	11.1	86	1.55	11.9	25.43	0.12	1.5
40	M	28.995684	4.9374	262.3194	363.7485	1.905316	9.32	8.19	87.9	0.78	8.4	4.26	0.07	1.5
41	M	37.180784	5.064	577.4056	817.551	1.509064	3.42	1.82	53.1	1.04	30.4	11.19	0.06	2.66
42	M	26.469996	12.1536	257.0504	393.273	1.795246	18.76	16.26	86.7	1.28	6.8	33.24	0.16	17.83
43	M	31.229047	4.8108	532.0922	586.8225	1.310938	5.25	4.45	84.7	0.62	11.8	129.52	0.08	6.49
44	M	32.316496	4.9374	521.5542	606.5055	1.068784	4.91	3.07	62.5	1.24	25.3	1.22	0.06	4.56
45	S	36.946924	25.8897	262.3194	266.427	0.815623	16.73	14.71	87.9	0.84	5	3.53	0.05	67.42
46	M	27.534059	13.7994	126.3792	146.142	2.323582	5.62	4	71.2	1.18	21	1.76	0.06	3.5
47	M	16.940201	27.4722	260.2118	484.0335	2.675806	8.33	7.22	86.7	0.62	7.5	163.26	0.38	36.81
48	M	10.509051	6.7098	192.7686	394.3665	3.303205	7.33	4.63	63.2	2.12	28.9	2.04	0.06	2.73
49	M	11.853746	5.1906	403.5286	534.3345	2.642785	3.36	2.34	69.6	0.68	20.1	47.66	0.1	37.04
50	S	31.766925	13.4829	674.3552	885.348	1.828267	2.8	1.81	64.6	0.76	27.1	156.56	0.3	7.23
51	M	17.653474	8.3556	115.8412	164.7315	2.840911	6.35	4.85	76.3	0.86	13.6	19.96	0.1	16.39
52	M	15.583813	6.0135	580.567	574.794	2.378617	2.6	1.53	58.7	0.86	33.2	1.13	0.05	35.68
53	S	28.118709	64.566	391.9368	449.0415	4.183765	10.47	7.55	72.1	2.1	20.1	4.44	0.05	3.9
54	S	37.940829	7.2795	588.9974	671.022	6.4402	9.83	8.96	91.1	0.6	6.1	68.98	0.11	9.18

55	M	37.08724	6.963	266.5346	253.305	3.754492	6.71	5.83	86.9	0.44	6.5	5.79	0.06	4.76
56	S	36.338888	5.1906	298.1486	307.98	3.281191	10.2	8.25	80.9	1.39	13.6	13.42	0.14	10.42
57	S	17.185754	37.4103	797.6498	899.5635	5.086339	12.68	8.62	68	3.06	24.1	52.5	0.6	28.27
58	S	28.492885	34.8783	548.953	758.502	5.647696	8.55	6.3	73.7	1.38	16.1	23.52	0.14	14.97
59	M	32.234645	4.9374	547.8992	629.469	1.674169	4.06	2.44	60	1.29	31.7	0.34	0.08	1.5
60	M	35.625615	27.0924	560.5448	684.144	2.642785	4.72	2.32	49.1	1.79	38	1.11	0.04	1.5
61	S	34.374464	5.8236	340.3006	441.387	4.205779	12.13	10.4	85.7	0.53	4.4	48.35	0.17	25.72
62	S	17.022052	16.6479	767.0896	452.322	2.851918	5.86	3.94	67.2	1.3	22.1	69.97	0.05	53.06
63	S	36.748143	12.2802	957.8274	1005.633	8.465488	8.62	7.88	91.4	0.59	6.8	208.36	0.94	12.63
64	S	3.236005	41.8413	665.9248	830.673	7.783054	5.63	5.21	92.5	0.25	4.4	11.58	0.06	2.62
65	S	34.105525	5.8869	495.2092	362.655	2.752855	6.78	5.59	82.4	0.73	10.7	1.92	0.07	2.12
66	M	34.608324	5.697	201.199	199.7235	2.752855	12.62	6.65	52.7	5.02	39.8	1.18	0.09	18.4
67	M	37.578346	9.2418	571.0828	467.631	5.460577	5.28	2.35	44.6	2.29	43.3	0.89	0.07	2.06
68	M	1.832845	9.9381	507.8548	482.94	4.965262	3.64	2.04	56.1	1.11	30.5	28.48	0.19	10.39
69	S	21.582322	4.7475	865.093	650.2455	4.45894	3.47	3.27	94.1	0.11	3.2	192.05	0.14	14.67
70	M	24.961599	5.0007	485.725	462.1635	1.013749	5.68	4.91	86.4	0.68	12	23.5	0.04	1.5
71	S	37.473109	47.475	52.6132	687.987	4.249807	7.86	6.78	86.3	0.61	7.8	122.86	0.19	67.04
72	S	32.655593	5.4438	662.7634	605.412	4.194772	6.9	6.1	88.4	0.48	7	2.29	0.12	10.87
73	S	36.081642	8.2923	617.45	418.4235	5.218423	15.1	13.79	91.3	0.69	4.6	34.96	0.14	11.66
74	S	38.315005	24.1806	770.251	574.794	7.98118	10.6	6.76	63.8	1.02	9.6	25.84	0.31	1.5
75	S	35.730852	14.1159	688.0546	538.7085	3.622408	12.39	10.72	86.5	0.98	7.9	7.06	0.03	1.5
76	M	35.040965	5.0007	371.9146	263.1465	2.873932	5.88	4.67	79.5	0.96	16.3	19.59	0.1	8.27
77	M	13.525845	7.1529	668.0324	680.8635	4.040674	15.93	15.15	95.1	0.33	2.1	0.2	0.02	10.2
78	S	40.829	41.4615	710.1844	555.111	7.849096	16.4	14.87	90.7	0.92	5.6	6.81	0.2	1.5
79	S	35.68408	47.9814	811.3492	745.38	10.435741	17.04	15.69	92.1	0.58	3.4	30.39	0.52	22
80	S	40.829	16.0149	1068.4764	661.1805	3.886576	7.12	5.98	84	0.98	13.7	10.59	0.07	42.3
81	S	5.223815	76.8462	889.3304	556.2045	5.878843	8.51	8.13	95.5	0.16	1.9	69.13	2.02	22.27
82	S	40.829	5.3172	1114.8436	885.348	8.432467	2.27	0.61	26.8	1.29	56.9	11.38	0.12	11.1

83	S	15.092707	4.8108	129.5406	131.9265	7.992187	11.06	9.22	83.4	1.02	9.2	7.56	0.12	2
84	M	39.624621	16.1415	792.3808	445.761	2.884939	4.77	3.12	65.5	1.16	24.3	100.46	0.07	29.6
85	S	37.917443	56.4636	579.5132	292.671	10.116538	11.96	11.31	94.6	0.5	4.2	74.17	0.23	50.27
86	S	39.6597	20.5725	839.8018	579.168	4.2388	8.73	7.87	90.2	0.31	3.6	0.69	0.03	3.42
87	M	34.783719	5.064	915.6754	584.6355	2.939974	14.12	12.11	85.8	0.52	10.5	81.63	0.4	3.17
88	S	33.415638	9.5583	906.1912	784.746	3.259177	7.33	5.41	73.8	1	13.6	92.61	0.04	6.69
89	M	20.494873	4.7475	18.8916	177.8535	3.369247	9.59	8.49	88.5	0.52	5.4	15.34	0.14	3.17
90	M	19.337266	5.4438	44.1828	324.3825	2.048407	6.6	3.93	59.6	2.1	31.8	0.93	0.06	1.71
91	M	22.576227	5.3805	44.1828	547.4565	2.279554	2.81	1.78	63.5	0.7	25	10.85	0.07	15.5
92	S	7.27009	31.017	57.8822	597.7575	5.900857	5.27	4.93	93.5	0.13	2.4	10.44	0.04	7.21
93	S	30.679476	24.687	54.7208	557.298	4.866199	6.52	5.99	91.8	0.43	6.6	84.29	0.1	104.5
94	M	18.074422	7.9125	33.6448	502.623	2.543722	2.53	1.33	52.5	0.97	38.4	7.84	0.15	12.35
95	S	30.223449	41.145	15.7302	306.8865	3.149107	6.81	5.39	79.2	1.27	18.7	111.35	0.12	2.84
96	M	35.134509	15.8883	2.1844	552.924	5.11936	6.32	4.89	77.3	0.94	14.8	36.14	0.05	12.36
97	M	22.541148	9.8748	20.9992	407.4885	1.432015	8.39	6.97	83.1	1.15	13.17	11.53	0.04	1.5
98	S	35.087737	49.9437	89.4962	950.958	7.474858	4.98	3.18	63.9	1.41	28.4	149.26	0.12	102.54
99	M	34.725254	13.6095	42.0752	338.598	3.105079	5.23	3.36	64.2	0.73	13.9	57.77	0.06	85.54
